# Bacterial meningitis secondary to otogenic infection in 10 French bulldogs: A retrospective case series

**DOI:** 10.1002/vro2.63

**Published:** 2023-06-13

**Authors:** Sarah Butterfield, Danielle Whittaker, Joana Tabanez, Jordina Caldero Carrete, Clare Pitchford, Charles R. J. Mattias, Abbe Crawford, Clare Rusbridge

**Affiliations:** ^1^ Department of Clinical Science and Services Royal Veterinary College Hatfield UK; ^2^ Fitzpatrick Referrals Orthopaedics and Neurology Surrey UK; ^3^ VPG Exeter Exeter UK; ^4^ Wear Referrals Stockton‐on‐Tees UK

## Abstract

**Background:**

There is limited published information to guide the clinical management of bacterial meningitis/encephalitis in dogs.

**Methods:**

This was a retrospective case series comprising 10 French bulldogs from two referral centres. The cases were diagnosed with bacterial meningitis/encephalitis suspected secondary to otogenic infection based on detection of abnormal fluid/soft tissue opacity within the middle/inner ear, associated meningeal/intracranial involvement through MRI, the findings of cerebro‐spinal fluid (CSF) analysis suggestive of sepsis and/or clinical improvement following antibiosis.

**Results:**

Ten dogs were included (three female and seven male), with a median age of 60 months. Dogs presented with acute onset (median 2 days), progressive history of vestibular signs and/or intra‐oral or cervical pain. Five dogs had gross signs of concurrent otitis externa. Common MRI findings included material within the tympanic bulla with adjacent meningeal enhancement. Analysis of CSF documented pleocytosis in all eight dogs, intracellular bacteria seen in three with positive bacteriological culture in two dogs. One dog was euthanised following diagnosis. Nine remaining dogs received antimicrobial therapy and six underwent surgical management. Three dogs treated surgically were neurologically normal within 2 weeks and the remaining three improved. Two dogs treated medically improved and one had complete resolution reported within a 4‐week follow‐up period. Study limitations include its retrospective nature and small sample size with minimal longer term follow‐up.

**Conclusions:**

Bacterial meningitis/encephalitis in French bulldogs can require both medical and surgical treatment to achieve a favourable outcome.

## INTRODUCTION

Bacterial meningitis is rarely reported in companion animals and it is often considered a neurological emergency requiring rapid diagnosis and treatment. Several mechanisms of infection have been described, including haematogenous spread, direct spread from adjacent structures such as inner ear or frontal sinus, migration of foreign material, aberrant parasite migration or direct inoculation such as a bite wound.^1^ Otogenic infection has been uncommonly cited in the veterinary literature as the primary cause, including one previous case report^2^ and a subsequent case series describing the medical and surgical management of cats and dogs with complications of otitis media (OM)/otitis interna (OI).^3^, ^4^

Meningitis refers to inflammation of the meninges surrounding the brain and/or spinal cord, where both infectious and non‐infectious aetiologies are described. Meningoencephalitis describes the additional inflammation within brain parenchyma. Bacterial meningoencephalitis is well documented in humans following oto‐sinogenic infections, as well as described in adults as a consequence of cholesteatomas or polyps.^5^, ^6^ Acute infections that can progress to life‐threatening meningitis are more frequent in infants and juveniles, with diabetes mellitus and immuno‐suppression reported as predisposing factors.^5^, ^6^ The most common clinical signs reported in people include fever, headache, neck pain, vomiting and seizures.^5^, ^6^, ^7^, ^8^ Signs more commonly attributed to meningoencephalitis in animals include mentation change, proprioceptive deficits, paresis and cranial nerve deficits,^9^ either from a space‐occupying lesion or diffuse inflammation/oedema. In humans with focal empyema, rapid decompressive surgery and antimicrobial therapy based on bacteriological culture is considered the most successful treatment, often resulting in rapid and complete resolution of the clinical signs.^5^, ^6^, ^7^

The aim of this retrospective case series was to describe the clinical presentation, imaging findings, cerebro‐spinal fluid (CSF) analysis and outcome of 10 French bulldogs where otogenic infection was cited as the likely primary cause of bacterial meningitis/encephalitis.

## MATERIALS AND METHODS

Medical records were obtained from two referral centres for the period January 2018 to January 2021. The databases were searched to identify French bulldogs that had been diagnosed with OM/OI with intracranial involvement following MRI and CSF analysis. To be included in this study, dogs needed to have undergone MRI that documented middle/inner ear effusion, either with contrast enhancement of adjacent structures including the meninges or CSF analysis indicative of bacterial infection (neutrophilic pleocytosis or intracellular bacteria detected on cytological evaluation) with or without positive bacterial culture.

Information collected from the case records included signalment, clinical history and concurrent conditions, physical characteristics including otoscopic examination, neurological examination, MRI findings, CSF analysis (including total protein concentration [TP], total nucleated cell count [TNCC] and cytological evaluation), CSF bacteriological culture and sensitivity results, antibiotic treatment initiated, whether surgery was performed, details of surgery undertaken, postoperative management and outcome.

The CSF analysis, including extended bacteriological culture, used reference intervals defined as total protein (<25 mg/dL) and TNCC less than 5 cells/µL.

MRI was performed using a 1.5 Tesla scanner (Siemens Symphony in dogs 1–6; Intera Philips Medical Systems, Eindhoven, The Netherlands, in dogs 7–10), and sequences obtained included T2‐weighted (T2W), T2W fluid attenuation inversion recovery and T1‐weighted (T1W) sagittal, transverse and dorsal views with/without T1 post‐gadolinium contrast images (0.2 mL/kg as a single bolus of 279.32 mg/mL Dotarem solution in dogs 1–6; 0.1 mL/kg of 1.0 mmol/mL Gadovist solution in dogs 7–10).

All dogs underwent concurrent otoscopic examination. Additional diagnostic testing, including myringotomy, culture and histopathology of excised tissue following surgery, was performed on an individual case basis. Cytology of samples from the external ear canal was performed in one case. Bacterial culture was performed on swab samples from the external ear canal in three cases, from fluid obtained via myringotomy in one dog and from fluid/tissue samples obtained during surgery in four dogs. Histopathology was performed on tissue removed during surgery in one dog.

The short‐term outcome was determined by repeat examination or by telephone consultation with the owner or referring veterinarian within at least 4 weeks of discharge.

## RESULTS

### Clinical findings

Ten dogs were included in the study (Table 1), with a median age of 60 months (range 24–133 months). Five were male neutered, two male entire and three female neutered. Clinical signs were predominantly acute in onset, with a median duration of 2 days prior to presentation, and ranged from 1 to 7 days in nine dogs and 60 days in one dog (where signs were predominantly pain on opening the mouth). Neurological signs were progressive in all dogs. Six dogs had a previous history and clinical signs compatible with a diagnosis of atopic dermatitis made by the primary care veterinary surgeon and requiring long‐term medical management. Five dogs had previously documented otitis externa (OE) from the referring veterinary surgeon's medical records.

**TABLE 1:** Signalment, clinical summary, treatment and outcome of 10 French bulldogs with bacterial meningitis secondary to otogenic infection.

Dog	Sex	Onset (days)	Neurological examination	Neuro‐anatomical localisation	Other clinical features	MRI features	Medical treatment	Surgery	Hospitalisation (days)	Outcome
1	Male entire	1	Vestibular ataxia with falling to the left, left head tilt, horizontal nystagmus with a fast phase to the right, left facial paralysis, postural reaction deficits in both pelvic limbs, worse on the left.	Left vestibular (central), left facial neuropathy	i. Neutrophilia, monocytosis ii. Conjunctival hyperaemia left eye on presentation iii. Previous history of right otitis externa iv. Chronic dermatitis, suspected atopy v. Left external ear swab cultured a heavy growth of coagulase positive *Staphylococcus*	i. T2W hyperintense material within left tympanic bulla, rim of contrast enhancement, thickening of left external ear canal integument with contrast enhancement of ear canal walls. ii. Diffuse contrast enhancement of adjacent meninges extending from the frontal lobe to the caudal aspect of the cerebellum, as well as of the left facial nerve. iii. Ill‐defined T2W hyperintensity and contrast enhancement of the left medial pterygoid muscle and left ventral aspect of the temporal muscle.	i. Amoxicillin‐clavulanate (15 mg/kg twice daily per os (PO) for 11 weeks). ii. Prednisolone (0.4 mg/kg once daily for 5 days, followed by 0.2 mg/kg once daily for 5 days followed by 0.2 mg/kg every other day, 5 more days).	No	3	Improved. Left head tilt and facial paralysis at 4‐week re‐examination. Improved but persistent left head tilt and left facial paresis at 10‐week re‐examination.
2	Female neutered	1	Vestibular ataxia with falling to the left, left head tilt, horizontal nystagmus with fast phase to right.	Left vestibular (peripheral)	i. Neutrophilia ii. Elevated alanine aminotransferase iii. C3–C4 ventral slot performed 3 days previously iv. Left ear swab cultured *Escherichia coli*	T2W hyperintense material within left tympanic bulla with contrast enhancement of this and adjacent meninges.	i. Cefuroxime (20 mg/kg four times daily IV) and metronidazole (10 mg/kg twice daily IV for 72 h). ii. Cefalexin (22 mg/kg twice daily for 7 weeks).	No	5	Improved with persistent mild vestibular signs at 3 week re‐examination
3	Male neutered	7	Stiff short‐strided gait, low head carriage, severe neck pain.	Cervical hyperaesthesia	i. Neutrophilia ii. Elevated C‐reactive protein iii. Left retropharyngeal mass, chemodectoma, bilateral otitis externa: receiving oclacitinib	i. Bilateral T2W hyperintense material within tympanic bulla. ii. Left‐sided supra‐collicular fluid accumulation, ventriculomegaly (likely congenital), cervical syringomyelia, degenerative lumbosacral disc disease.	i. Cefuroxime (22 mg/kg four times daily IV). ii. Methadone (0.2 mg/kg every 4 h IV). iii. Dexamethasone (0.5 mg/kg once daily IV). iv. Levetiracetam (30 mg/kg bolus, followed by 20 mg/kg four times daily IV). v. Phenobarbitone (loading dose of 18 mg/kg given in 6 mg/kg increments every 4 h). vi. Ketamine continuous rate infusion (10 µg/kg/min). vii. Propofol infusion (0.2 mg/kg/min). viii. Mannitol (1 g/kg IV over 20 min).	No	Euthanased	Euthanased after status epilepticus.
4	Female neutered	6	Left head tilt, horizontal nystagmus, left miosis, left facial paralysis, left corneal ulcer, paraparesis, absent postural reactions in pelvic limbs, cutaneous trunci cut‐off T11.	Left vestibular (peripheral), left facial neuropathy, T3–L3 myelopathy	i. Epistaxis, rhinitis with upper airway obstruction, tracheostomy tube with incidental pneumomediastinum ii. Myringotomy of left ear with *Staphylococcus* cultured	i. T2W hyperintense material within left tympanic bulla with contrast enhancement of this, left facial and vestibulocochlear nerve. ii. Para‐aural cellulitis and contrast enhancement. iii. Right ill‐defined T2W/FLAIR hyperintense piriform lobe lesion with no associated contrast enhancement. iv. Mediastinal lymphadenopathy and incidental pneumomediastinum/thorax. T10 ‘butterfly’ vertebra with mild spinal cord compression. v. L7–S1 intervertebral disc protrusion.	i. Cefalexin (27 mg/kg twice daily PO for 2 months). ii. Marbofloxacin (1.85 mg/kg once daily PO for 2 months). iii. Prednisolone (0.4 mg/kg every other day PO for 2 months).	No	7	Improved. Mild head tilt and normal CSF at 4‐week re‐examination. All signs resolved by 8 weeks.
5	Male neutered	60	Pain opening mouth and palpating left ear, temporalis muscle atrophy.	Temporomandibular joint/aural pain	i. Chronic dermatitis, atopy, pyoderma, recurrent bilateral otitis, receiving prednisolone (iatrogenic Cushing's signs) ii. BOAS and right TECA surgery	i. Bilateral T2W hyperintense material within tympanic bulla with contrast enhancement of left side. ii. T2W focal hyperintense lesion with contrast enhancement ventral to the left bulla continuous with the ear canal. iii. Meningeal contrast enhancement at level of left brainstem/trigeminal nerve root. iv. Para‐aural cellulitis and contrast enhancement of left temporalis and medial pterygoid muscle.	Marbofloxacin (2 mg/kg once daily PO for minimum 1 week).	Left TECA and ventral bulla osteotomy 1 week following diagnosis.	2	Neurologically normal 2 weeks after surgery.
6	Male neutered	1	Vestibular ataxia, right head tilt, rotary nystagmus, right ventrolateral strabismus, right levator angularis ocular muscle fasciculation, tonic–clonic seizure.	Right vestibular (central) + forebrain	i. Elevated C‐reactive protein ii. Chronic dietary responsive allergic skin disease, managed with oclacitinib	T2W hyperintense material within right tympanic bulla with contrast enhancement of this, the right cochlea and adjacent meninges from medulla to pons.	i. Cefuroxime (22 mg/kg three times daily IV), then cefalexin (21 mg/kg three times daily PO for 30 days). ii. Marbofloxacin (2 mg/kg once daily IV then 2 mg/kg once daily PO for 30 days). iii. Metronidazole (10 mg/kg twice daily IV then 9 mg/kg twice daily PO for 30 days).	Right TECA and LBO 3 months following initial diagnosis, Left TECA and LBO 3 months after.	9	Improved with residual head tilt. Recurrence of left para‐aural abscessation 4 months following left TECA/LBO surgery treated medically.
7	Female neutered	3	Obtunded mentation, right head tilt, horizontal and rotary nystagmus with fast phase to the left, reduced menace response and lateral palpebral reflex of left eye.	Right vestibular (central), right facial neuropathy	i. Toxic neutrophils, elevated C‐reactive protein ii. Arrhythmia (supraventricular premature complexes, tachycardia and ventricular premature complexes) iii. Previous bilateral TECA surgery, daily oclacitinib and hypoallergenic diet for dermatitis iv.	i. Poorly defined T2W/FLAIR intra‐axial hyperintensity within right caudal aspect of the pons. ii. T2W hyperintense material within tympanic bulla, rim of contrast enhancement. iii. Bilateral thickening and irregular bulla walls. iv. Para‐aural cellulitis and contrast enhancement. v. Marked enhancement and thickening of the adjacent meninges.	i. Cefalexin (20 mg/kg three times daily 14 days). ii. Paracetamol (10 mg/kg twice daily 7 days). iii. Gabapentin (10 mg/kg three times daily 14 days).	Right LBO, culture of bulla lavage fluid *Pseudomonas aeruginosa*.	9	Neurologically normal on examination by referring veterinarian 2 weeks after surgery.
8	Male entire	1	Vestibular ataxia, right head tilt, horizontal and rotary nystagmus.	Right vestibular	i. Single episode haematemesis ii. Elevated C‐reactive protein iii. Previous history of right otitis externa and corneal ulcers iv. Atopy on examination, fed hypoallergenic diet	i. T2W hyperintense material within tympanic bulla, rim of contrast enhancement. ii. Contrast enhancement and thickening of meninges, right medulla oblongata, cerebellar hemisphere, vestibulocochlear nerve and facial nerve. iii. Para‐aural cellulitis and contrast enhancement.	Cefalexin (19 mg/kg twice daily PO) for 3 weeks.	Right TECA and LBO Ear and bulla swabs isolated *Staphylococcus pseudintermedius*.	5	Improved by 3 week telephone consultation.
9	Male neutered	1	Vestibular ataxia, right head tilt, horizontal and rotary nystagmus with a fast phase to the left, right facial paralysis, right ventrolateral strabismus.	Right vestibular , right facial neuropathy	i. Single episode vomiting ii. Previous history otitis externa	i. Bilateral T2W material within tympanic bullae, with thickened bullae wall and rim contrast enhancement, more on right. ii. Contrast enhancement of right cochlea and content of right facial canal. iii. Meningeal enhancement along right side of brainstem, right rostral cerebellar peduncle and right cerebellar hemisphere. iv. Associated extra‐axial brainstem compression with intra‐axial T2W hyperintensity of affected brain parenchyma v. Para‐aural cellulitis and contrast enhancement. vi. Temporal bone contrast enhancement suggestive of osteomyelitis.	Cefalexin (28 mg/kg twice daily for 6 weeks).	Right TECA and LBO. Right temporary tarsorrhaphy Bullae swab isolated *Staphylococcus pseudintermedius*.	5	Deemed neurologically normal by owner telephone update 2 weeks after surgery.
10	Female neutered	3	Vestibular ataxia with drifting to the right, right head tilt, right facial paresis, horizontal nystagmus with fast phase to left, right positional ventrolateral strabismus.	Right vestibular (peripheral), right facial neuropathy	i. Chronic atopy managed with oclacitinib and lokivetmab injections ii. Neutrophilia, lymphopenia iii. Mild non‐specific hepatopathy iv. Markedly elevated C‐reactive protein	i. T2W hyperintense material in right tympanic bulla with contrast‐enhanced lining. ii. Marked contrast enhancement of the tissues within the right facial canal, right trigeminal nerve and meningeal enhancement along the adjacent ventrolateral surface of brainstem. iii. Para‐aural cellulitis and contrast enhancement. iv. Material in left bulla (contralateral).	Amoxicillin‐clavulanate (20 mg/kg twice daily PO for 3 weeks).	i. Right TECA–LBO. ii. Severe chronic diffuse dermal fibrosis with multifocal sebaceous gland loss histopathology. iii. Culture abscessated tissue during surgery and middle ear *Staphylococcus. pseudintermedius*.	9	Swelling at lateral aspect of the pinna, 3 weeks post‐op, treated by referring veterinarian and lost to follow‐up.

Abbreviations: BOAS, brachycephalic obstructive airway syndrome; CSF, cerebro‐spinal fluid; ; FLAIR, fluid attenuation inversion recovery; FN, female neutered; IV, intravenous; LBO, lateral bulla osteotomy; TECA, total ear canal ablation; T2W, T2‐weighted.

Abnormalities identified on general physical examination were visible OE with otoscopic examination (5/10), corneal ulceration associated with ipsilateral facial nerve paralysis (*n* = 1), corneal keratitis (*n* = 1) and pain on opening the mouth (*n* = 1). One dog showed signs consistent with iatrogenic hyperadrenocorticism (abdominal distention, generalised muscle wastage and dermal changes) following chronic steroid therapy for atopic dermatitis and one dog had a well‐healing incision site from a ventral slot procedure performed 3 days previously for management of a C3–C4 intervertebral disc extrusion. One dog had multiple co‐morbidities, including epistaxis and rhinitis, that were deemed unrelated to the current presentation and under investigation. The same dog had developed upper respiratory obstruction requiring temporary tracheostomy tube placement. Two of the dogs that had evidence of chronic OE had previously undergone surgical management by total ear canal ablation (TECA) procedure, bilaterally in one dog and unilaterally in the other (contralateral to presenting affected ear). Pyrexia was not detected in any dog. One dog was found to have an arrhythmia with a structurally normal heart confirmed by echocardiography and no treatment was deemed necessary.

On neurological examination, abnormalities included obtundation (*n* = 2), head tilt (*n* = 8), rotatory nystagmus (*n* = 4), horizontal nystagmus (*n* = 7), ventrolateral positional strabismus (*n* = 3), ipsilateral miosis deemed secondary to the concurrent corneal ulceration (*n* = 1), facial paralysis (*n* = 5), temporalis muscle atrophy (*n* = 1), low head carriage (*n* = 1), neck pain (*n* = 1) and pelvic limb postural reaction deficits (*n* = 2, one of which had a suspected concurrent T3–L3 myelopathy). One dog presented with a history of seizures suspected secondary to the condition; another dog developed seizures following diagnosis. Out of 10 dogs, the neuro‐anatomical localisation was defined as right vestibular syndrome in five dogs (two of which had evidence of central involvement), left vestibular syndrome in three dogs (one of which had signs of central involvement) and neurologically normal with pain in two dogs (cervical hyperaesthesia and pain on opening the mouth). Horner syndrome was not a clinical feature in any dog.

Haematology and serum biochemistry were performed in eight of 10 dogs. Abnormalities documented were neutrophilia (*n* = 5), monocytosis (*n* = 1), lymphopenia (*n* = 1) and elevated alanine aminotransferase (*n* = 2). Additionally, C‐reactive protein (CRP) was assessed in seven cases and was elevated above the reference interval in five cases (RI: 0 – 10 mg/L, median 30.5 mg/L, range 19.2–116.8 mg/L). Additional blood tests included creatine kinase/aspartate aminotransferase (*n* = 1), anti‐2M myofibre antibodies (*n* = 1) and Toxoplasma/Neospora serology (*n* = 2), all of which were within normal limits.

### MRI imaging and CSF findings

**FIGURE 1:**
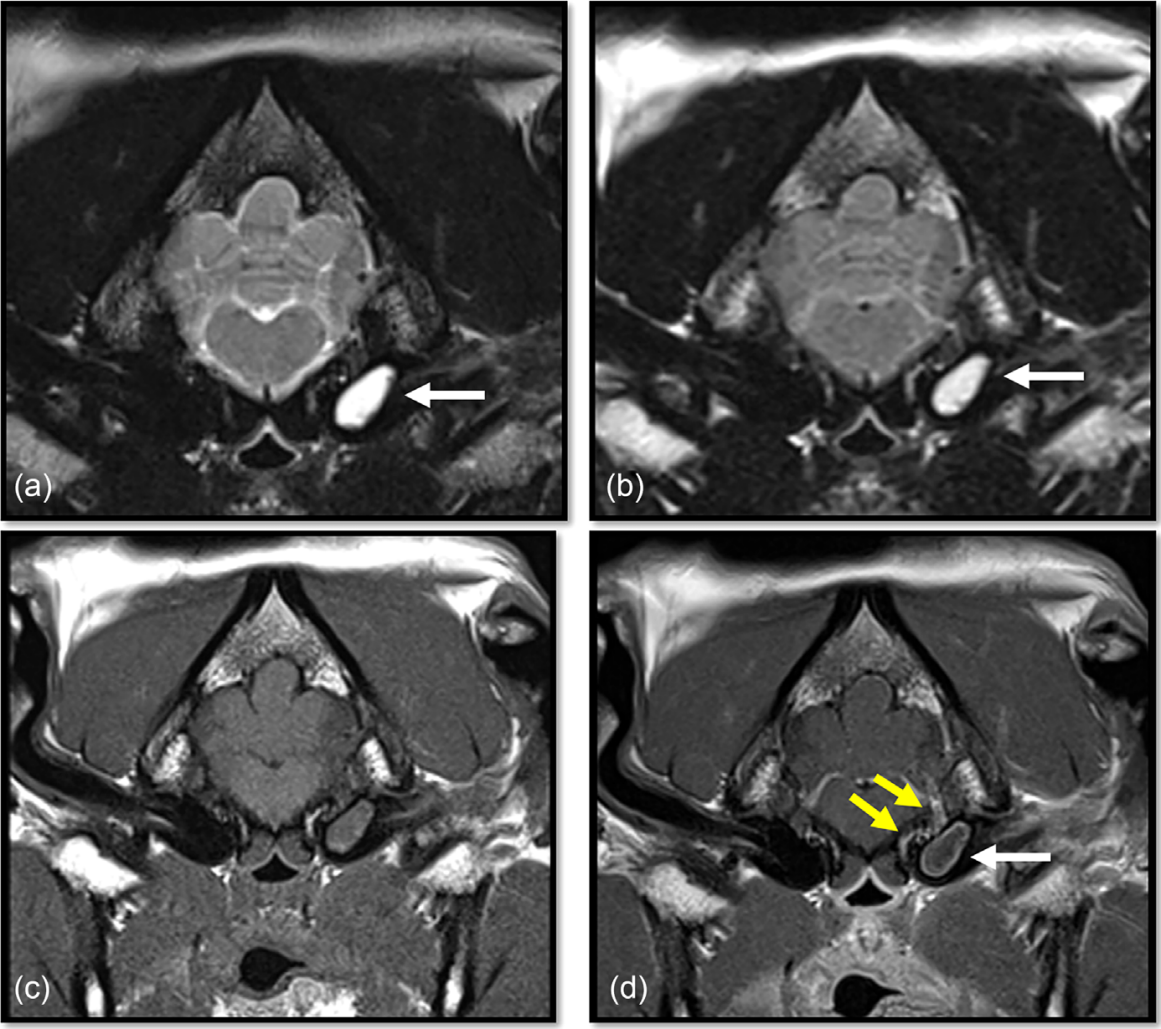
Representative transverse MR images of a French bulldog with bacterial meningitis secondary to otogenic infection. MRI sequences include T2‐weighted (T2W) transverse (a), T2W fluid attenuation inversion recovery (FLAIR) transverse (b), T1 transverse pre‐gadolinium contrast (c) and T1 transverse post‐gadolinium contrast administration (d) at the level of the tympanic bullae. Material confined to the bulla is seen within the left middle ear (white arrows) that is hyperintense on T2W and FLAIR images and isointense on T1‐images compared to normal grey matter. In (d), the material within the left bulla shows a ring of contrast enhancement as well as enhancement of the adjacent meninges (yellow arrows).

**FIGURE 2:**
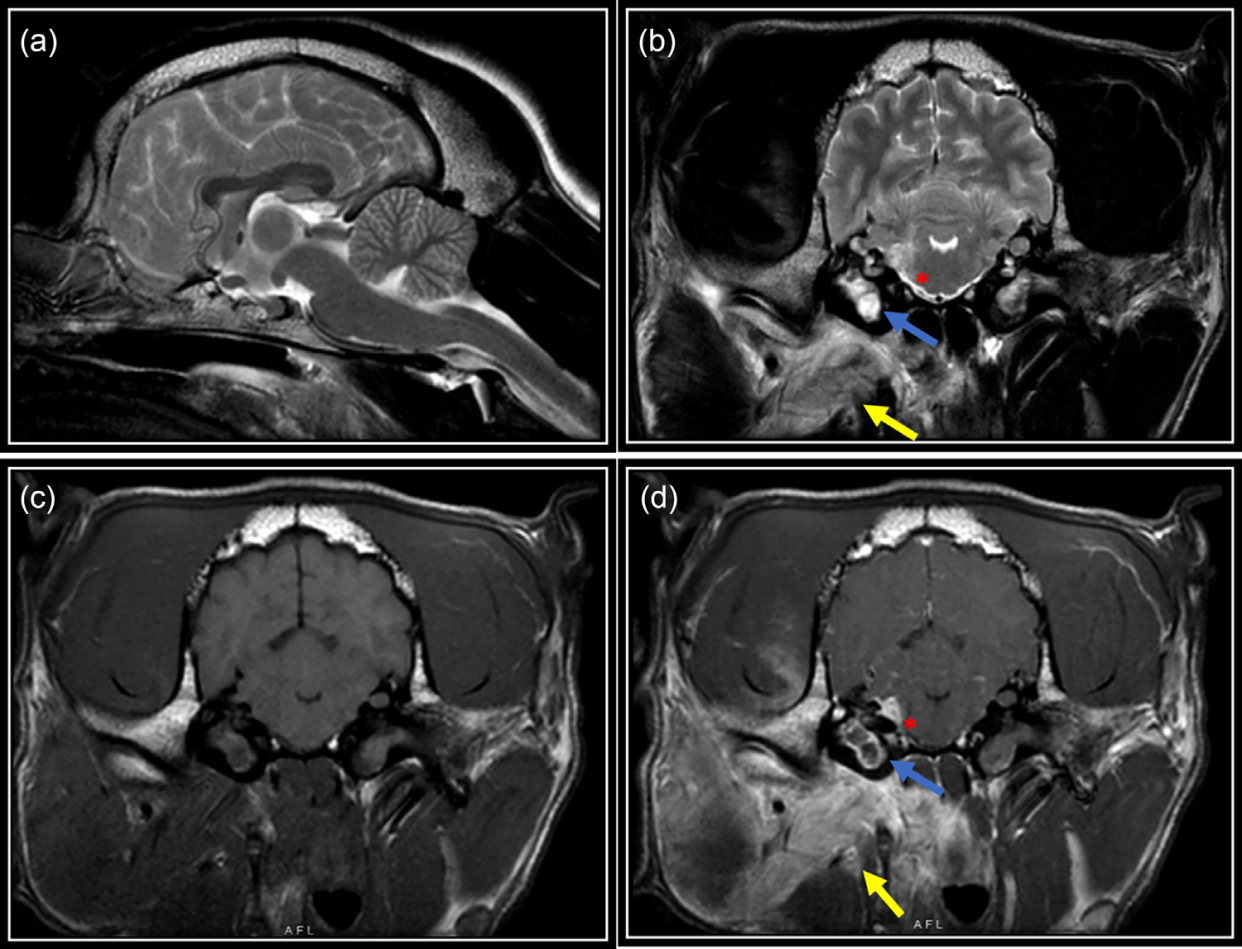
Representative MR images from case 9 including T2‐weighted (T2W) sagittal (a), T2W transverse (b), T1 transverse pre‐gadolinium contrast (c) and T1 transverse post‐gadolinium contrast administration (d) at the level of the tympanic bullae. Well‐defined material is seen within the right middle ear (blue arrows) that is hyperintense on T2W images, isointense on T1‐images compared to normal grey matter with a ring of peripheral contrast enhancement. T2W hyperintense, T1W isointense minimally contrast enhancing material is also present within the left tympanic bulla. Note the marked diffuse T2W hyperintensity of the para‐aural soft tissue (b, yellow arrow) and heterogenous contrast enhancement of these structures (d, yellow arrow). Focal intra‐axial T2W hyperintensity is also seen of the adjacent brainstem and meninges, with homogenous contrast enhancement at this level (red asterisk).

MRI of the head was performed in all cases, with administration of gadolinium contrast agent in nine dogs (Figures 1 and 2). T2W hyperintense material within the tympanic bulla was seen in all 10 cases, with heterogenous T1W post‐gadolinium contrast enhancement of the material in all. In addition, contrast enhancement was detected in the tympanic bulla wall lining (*n* = 3), adjacent meninges (*n* = 8), vestibulocochlear nerve (*n* = 3), facial nerve (*n* = 5), trigeminal nerve root (*n* = 2), cochlea (*n* = 1), intra‐axial lesions/contrast enhancement suggestive of meningoencephalitis (*n* = 5), cerebellum/peduncles (*n* = 3), adjacent soft tissue consistent with para‐aural cellulitis (*n* = 5), temporal bone suggestive of osteomyelitis (*n* = 1) and regional lymph nodes (*n* = 3). In four dogs, there was also T2W hyperintense material present within the contralateral clinically unaffected tympanic bulla.

Additional MRI findings included supra‐collicular fluid accumulation and ventriculomegaly (*n* = 2), cervical syringomyelia (*n* = 1), an ill‐defined T2W hyperintense piriform lobe lesion (*n* = 1) in a dog without a history of seizure activity, pneumomediastinum/pneumothorax secondary to tracheal tube placement in the dog with rhinitis (*n* = 1) and a compressive L7–S1 intervertebral disc protrusion (*n* = 1). Additional CT of the head, thorax and abdomen performed in dog 3 documented several mass lesions, including a left retropharyngeal mass, regional lymphadenopathy, heart base mass (most consistent with chemodectoma) and a solitary splenic nodule.

**FIGURE 3:**
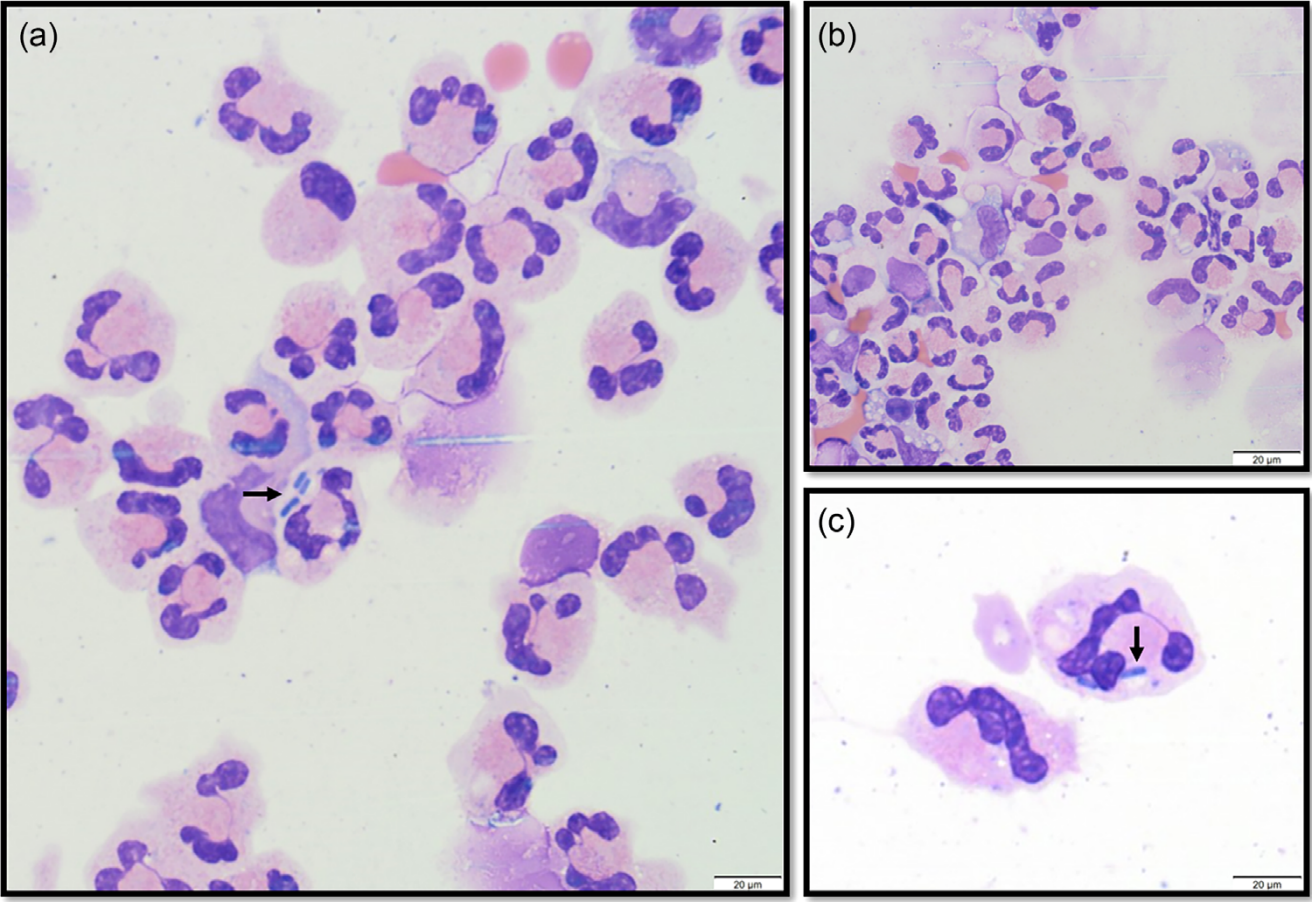
Representative cerebrospinal fluid (CSF) sample with two EDTA (a and b) and one plain tube (c) shown from dog 2 with Modified Wrights stain. In all three images, there is a marked neutrophilic pleocytosis with basophilic intracellular rods visible (black arrows) within neutrophils. Despite the presence of visible bacteria, culture of the CSF was negative.

**FIGURE 4:**
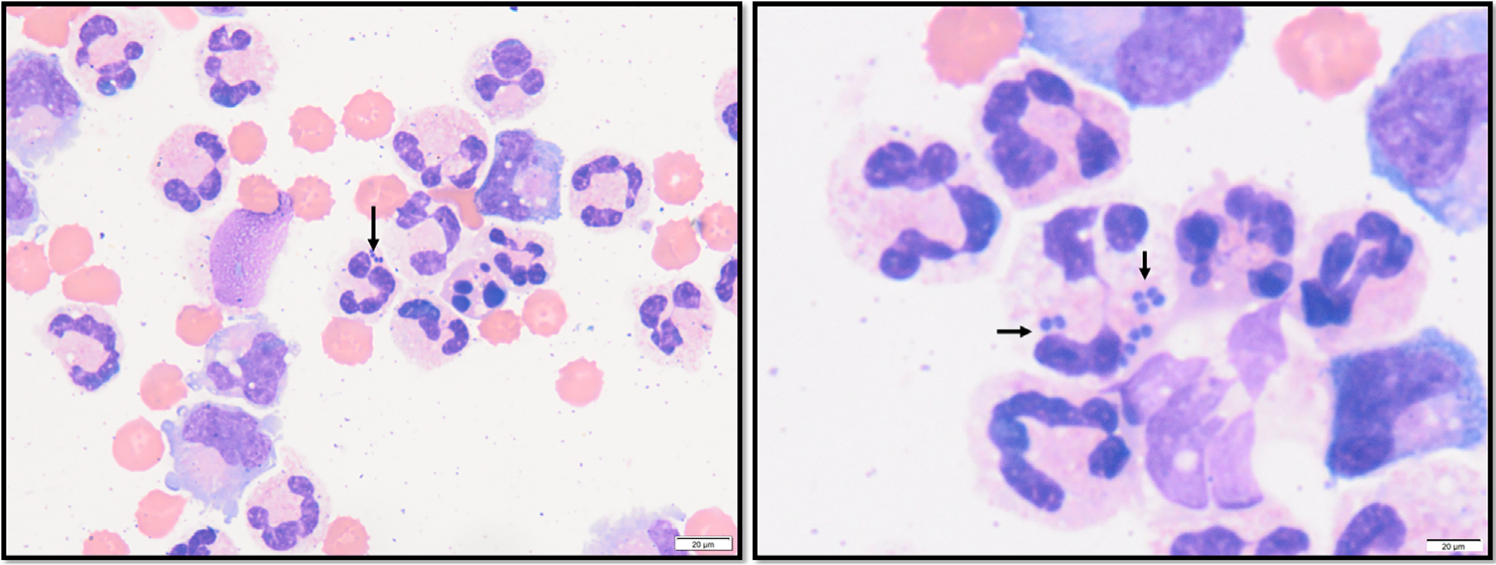
Representative cerebrospinal fluid (CSF) sample from dog 8 stained with haematoxylin and eosin. In both images, there is a marked neutrophilic pleocytosis with basophilic intracellular cocci visible (black arrows) within neutrophils. Despite the presence of bacteria, culture of the CSF was negative.

Cerebro‐spinal fluid was collected in eight of 10 dogs; seven taken from the cerebellomedullary cistern and one lumbar sample (Table 2). In the two cases where CSF was not performed, this was due to prompt surgical intervention, one being maintained under general anaesthesia following imaging and one the following day; consequently, obtaining CSF was deemed unnecessary. All samples were sent for cytological assessment, which revealed mild to marked elevations in total protein (*n* = 7, median 135.2 mg/dL, range 25.5–1582.6 mg/dL), elevated TNCC in all sampled dogs (*n* = 8, median 520/µL, range 10–1580/µL), neutrophilic pleocytosis (*n* = 7, range 65%–85%) and mixed mononuclear pleocytosis (*n* = 1). Intracellular rods were identified in one case (Figure 3) and intracellular cocci were identified in two cases (Figure 4). Despite visible infectious organisms, all three samples were negative for bacterial growth on culture. Culture of CSF yielded bacteria in two cases (dogs 3 and 8) despite no visible organisms on cytological evaluation, with coagulase‐negative *Staphylococcus* spp. and coagulase‐positive *Staphylococcus* spp. isolated following enrichment culture. Toxoplasma and Neospora PCR performed on CSF in one case was negative.

**TABLE 2:** Summary of cerebrospinal fluid (CSF) analysis performed in eight dogs with bacterial meningitis secondary to otogenic infection.

Dog	CSF cytology findings	Total protein (mg/dL)	Total nucleated cell count (/µL)	CSF culture
1	Marked non‐degenerate neutrophilic pleocytosis (70% neutrophils), large mononuclear cells (27%), small lymphocytes (3%), small lymphocytes. No infectious agents seen	106	1580	Positive for coagulase positive Staphylococcal species
2	Neutrophilic pleocytosis (74%), mononuclear cells (25%), eosinophils (1%). Intracellular rods seen	196.4	110	No growth
3	Neutrophilic pleocytosis (85%). Bacterial cocci seen	1582.6	400	No growth
4	Neutrophilic pleocytosis (66%), monocytes (13%), lymphocytes (2%), eosinophils (2%)	25.5	10	Positive for coagulase‐negative Staphylococcal species
5	Mononuclear pleocytosis	148	17	No growth
6	Neutrophilic pleocytosis (66%), large mononuclear cells (33%), small mononuclear cells (1%)	141.4	1440	No growth
7	Marked non‐degenerate neutrophilic pleocytosis (65%), monocytes (19%), lymphocytes (15%)	129	1495	No growth
8	Non‐degenerate neutrophilic pleocytosis (70%), lymphocytes (4%), monocytes (16%), macrophages (12%). Intracellular individual and paired cocci seen	44	640	No growth

Cytology of the external ear canal performed in one dog revealed pyogranulomatous inflammation, abundant cocci and Malassezia of the affected side. Bacterial culture and sensitivity performed on a swab obtained during surgery of the same dog documented growth of *Staphylococcus pseudintermedius*. Culture of the external ear was not performed in this case due to financial concerns.

An external ear swab yielded a heavy growth of *Escherichia coli* in one dog with rods seen on CSF cytology. One external ear canal swab cultured a heavy growth of coagulase‐positive *Staphylococcus* in the dog with positive CSF culture of the same species. The third case where culture of an external ear canal swab was undertaken revealed a growth of *S. pseudintermedius* where growth of the same species was cultured from samples obtained during surgery. All three dogs had clinical signs suggestive of OE on examination. A myringotomy sample yielded a scanty growth of coagulase‐positive *Staphylococcus* spp. in one dog. In the cases that underwent surgical management, culture of bulla lavage fluid at surgery documented *Pseudomonas aeruginosa* (*n* = 1) and *S. pseudintermedius* (*n* = 3). In nine of 10 cases, there was a positive bacterial culture by at least one means, which was the basis for antibiotic therapy.

### Treatment and outcome

All dogs were initiated on single or combination empirical intravenous (IV) antibiotic therapy, including amoxicillin‐clavulanate (*n* = 2), cefuroxime (*n* = 7), metronidazole (*n* = 2) and marbofloxacin (*n* = 2), prior to culture results (Table 1). Five dogs were continued on oral antibiotics including amoxicillin‐clavulanate (*n* = 2), cefalexin (*n* = 5) and marbofloxacin (*n* = 3), three of which were on combined therapy with more than one antibiotic. Excluding the dog that died during hospitalisation, the mean length of antibiotic treatment in all other dogs was 5 weeks (range 1–11 weeks), where the mean of those treated medically was 8.6 weeks in comparison to 3.2 weeks for surgically managed dogs.

Three dogs also received steroid therapy, including a single IV dose of dexamethasone (*n* = 1, 0.5 mg/kg) or a short course of oral prednisolone (*n* = 2, 0.4 mg/kg once a day or every other day). One dog developed seizures progressing to status epilepticus during hospitalisation. Interestingly, this dog was neurologically normal with cervical pain on presentation. Seizure activity continued despite treatment with levetiracetam (30 mg/kg IV bolus, followed by 20 mg/kg four times daily), phenobarbitone (loading dose of 18 mg/kg given in 6 mg/kg IV increments), ketamine (10 µg/kg/min) and propofol infusion (0.2 mg/kg/min). The dog also received mannitol (1 g/kg IV over 20 min) due to concerns regarding elevated intracranial pressure. Given the seizure severity and co‐morbidities documented on CT (left retropharyngeal and heart base mass), the dog was euthanised at the request of the owner.

Of the nine remaining dogs, all showed neurological improvement following initiation of antibiotic therapies. While not definitive, given the diagnoses made and in the absence of other specific treatments administered, this improvement was deemed likely to be a direct result of antibiotic therapy. Surgical intervention was initially declined in four cases due to varying factors, including age, presence of co‐morbidities, financial constraints and improvement following initiation of medical therapy. Two dogs had persistent vestibular signs (mild head tilt and ataxia) at 3‐ and 4‐week follow‐ups, respectively; the latter underwent repeat CSF sampling at this point, which was unremarkable. The owner then reported a complete resolution of neurological signs 8 weeks following initiation of treatment by telephone conversation. Another dog showed improvement, however it maintained a mild head tilt and facial paresis at both 4‐ and 10‐week re‐examinations. The fourth dog that was not operated on showed initial improvement following medical therapy and it underwent surgery 3 months later due to persistent vestibular signs. While an improvement was seen following the procedure, a subtle persistent right head tilt remained.

The other five dogs underwent surgical treatment of OM/OI consisting of a left (TECA) and ventral bulla osteotomy (*n* = 1), right lateral bulla osteotomy (LBO) (*n* = 1) or right TECA and LBO (n = 3). The specific surgical technique was performed at the discretion of the attending clinician. No surgical complications were reported and all five dogs showed neurological improvement during hospitalisation. Three dogs were deemed neurologically normal after 2 weeks of treatment by re‐examination (*n* = 1), by examination at the referring veterinary surgeon (*n* = 1) and by owner telephone consultation (*n* = 1). The two remaining cases improved with mild persistent vestibular signs; they were lost to follow‐up thereafter.

## DISCUSSION

Nine (90%) dogs in our case series presented with an acute onset of clinical signs between 1 and 6 days prior to presentation, all of which were progressive, consistent with previous reports.^1^, ^3^, ^4^ It has been suggested that onset of disease may affect outcome, with those presenting with acute (<24 h) or subacute (1–7 days) signs more likely to have a suppurative OM/OI and a poorer outcome.^3^ While the predominant clinical feature in eight (80%) dogs was vestibular dysfunction, in two dogs, only pain was identified.

Only five (50%) dogs had evidence of grossly visible OE on otoscopic examination, including three where external ear canal swabs revealed a positive bacterial culture. Swab samples from the external ear canal were not obtained in two dogs, one due to subsequent euthanasia. Furthermore, OM has been documented on MRI as an occult finding in 20% of dogs with chronic OE in one study,^10^ and another study reported OM in 50%–80% of chronic OE cases,^11^ raising the question of possible cause or consequence between OE and OM.

In agreement with previous reports, pyrexia did not appear to be a consistent finding,^1^, ^3^ with normal rectal temperature in all dogs. Horner syndrome was not a clinical feature in any dog; interestingly, by comparison, it was a common finding (33%) in a cat study.^4^

C‐reactive protein was assessed in seven cases and was elevated in five, indicating its use as a possible inflammatory biomarker. Further studies comparing its use in dogs with OM/OI without intracranial involvement would be required to validate this.

The MRI sequences showed T2W hyperintense material present within the tympanic bulla with varying degrees of contrast enhancement of this and surrounding structures. Meningeal enhancement on T1W post‐contrast imaging was found in 80% of the dogs, which has been documented in other veterinary studies.^1^, ^3^ Pathological bone changes have been described in middle ear disease with bulla thickening, lysis and/or periosteal proliferation or osteomyelitis of the petrous temporal bone,^12^ which was identified in one dog in this study. Of the three dogs presenting with central vestibular signs, two had focal intra‐axial lesions on MRI suggestive of meningoencephalitis; however, two of the five dogs presenting without central signs showed similar intra‐axial lesions. This suggests that examination alone is not enough to distinguish whether there is central involvement; therefore, MRI may be the preferable imaging modality even in cases without clear central vestibular system involvement on examination.

The two dogs presenting with pain only had the longest duration of clinical signs prior to presentation (7 and 60 days, respectively) and they displayed no evidence of intracranial involvement with MRI imaging. Furthermore, the dog with cervical pain only was documented to have intracellular bacteria on CSF, a markedly elevated CRP and progressed rapidly to status epilepticus within 12 hours of presentation. Therefore, it would seem that intra‐axial lesions are more likely in those with evidence of central involvement on clinical examination but that intracranial extension of an OM/OI cannot be excluded on MRI alone. While additional 3D MRI sequences such as constructive interference in steady state may aid in diagnosis and be of clinical use in humans when evaluating the middle/inner ear in greater detail, this is not typically performed in dogs and its use in evaluating the ear is isolated to a single experimental case series to date.^13^

Collecting CSF in cases of OM/OI to screen for meningeal/intracranial involvement should be considered to aid in the diagnosis, particularly where MRI findings alone are less supportive. No dogs included in the study underwent concurrent CT at the time of presentation and, therefore, it was not possible to make direct comparisons between CT and MRI; however, this might be a future study area of interest.

On CSF analysis, seven of eight dogs showed a predominantly neutrophilic pleocytosis. The differential diagnoses for a predominantly neutrophilic pleocytosis include suppurative inflammatory processes such as bacterial/fungal meningoencephalitis/meningoencephalomyelitis or steroid responsive meningitis–arteritis. In humans, it has been documented in acute inflammatory spinal cord disease such as trauma, haemorrhage, infarction and neoplasia.^14^

Bacteriological culture of the CSF was positive in two dogs, despite no visible bacteria on cytology. This shows discrepancy between the results of cytology and culture; even when infectious organisms are visible, it seems unlikely that a positive culture will be obtained. This is similar to previously documented poor success of CSF culture^1^, ^3^ with false‐negative results in approximately 70% of cases.^13^ Organisms that are typically implicated in veterinary cases with OM/OI include *P. aeruginosa* and *Staphylococcus intermedius*, as documented in our samples obtained from surgery, as well as *Streptococcus*, *Proteus*, *Klebsiella* and *E. coli* species.^3^, ^11^

The choice of antibiotic should be based on bacterial culture and sensitivity where possible. A positive culture result was more likely when sampling tissue during surgery rather than CSF. Antibiotics should be bactericidal and should achieve adequate concentrations within the CNS. Culture of the external ear canal or fluid obtained via myringotomy was successful (in 4/4 dogs) and therefore may be considered to guide antibiotic choice on the presumption that the bacterial colonies are the same. However, a difference between bacterial colonies between the external and middle ear has been reported in up to 89.5% of ears in dogs with OM.^15^

There are several limitations of this study, primarily its retrospective nature over a 2‐year period and the small study population. The differing approach of clinicians makes the treatment non‐standardised. The extent of clinical records varied between dogs, including that of the follow‐up, which varied in both time performed and nature (that is, in person re‐examination or telephone). Therefore, the follow‐up and outcome of the included dogs should be interpreted with caution. Additionally, repeat imaging was not performed during or after treatment to confirm visible improvement or resolution of infection, although repeat CSF sampling was performed in one case. This is not unusual in a clinical setting, where management is often dictated by treatment response, due to several factors including cost, requirement for repeating general anaesthesia and its associated risks. Also in those cases surgically managed, debridement and excision of infectious material may be deemed satisfactory at time of surgery; therefore, repeat imaging is not required.

With respect to the presence of OE and its role in disease development, the otoscopic examination of the external ear canals was performed in all of our cases. However, cytology samples from the external ear canal were not collected in all cases; ideally cytology samples should be included for further detail regarding the nature of the OE. Future studies, when dealing with dogs with suspected bacterial OM, should consider evaluation for the presence, severity and nature of concurrent OE (to include samples from the external canal for cytology and bacterial culture).

The French bulldog breed has gained significant popularity in recent years, becoming the most popular UK dog breed according to the 2019 Kennel Club census and second in 2021. As a brachycephalic breed, they may have abnormal pinnae and ear canal conformation as well as impaired auditory tube drainage due to nasopharyngeal abnormalities. Although bacterial meningitis remains an uncommon diagnosis, one case report exists describing a French bulldog^16^ and we describe here a cohort presenting with the condition. We suspect that their abnormal conformation might result in predisposition and that this pathology is more prevalent than previously thought. This could also be in part a reflection of increasing breed popularity. Comparative studies including other breeds are required to confirm this. Several reports have described French bulldog anatomy and how their brachycephalic conformation might predispose to ear disease. Comparison CT analysis of middle ear anatomy in dogs without clinical signs of ear disease found that brachycephalic dogs (specifically French bulldogs, English bulldogs and pugs) had significantly thicker tympanic bullae and smaller luminal volume in comparison to non‐brachycephalic dogs.^17^ In 36% of the brachycephalic dogs, they had material present within the tympanic cavity despite no clinical signs, compared to 0% of non‐brachycephalic dogs.^17^ Findings from our study support the theory that brachycephalic dogs have a greater prevalence of subclinical middle ear effusion, demonstrated by four dogs having abnormal material present within the contralateral tympanic bulla. Possible factors that could contribute to this include a thickened soft palate, reduced bulla volume leading to auditory tube dysfunction and inadequate bulla drainage.^17^ Another study supported the finding of reduced tympanic bulla volume in brachycephalic breeds, where 47% were documented to have middle ear effusion compared to 0% of mesaticephalic dogs.^18^ Specifically, in French bulldogs, 80% had abnormal fluid/soft tissue material present within the middle ear, as well as significantly more overlap between the tympanic bullae and the temporomandibular joint compared to other breeds, which may lead to deviation of the auditory tube and compromise drainage of the middle ear.^18^

The prognosis for bacterial meningitis/encephalitis secondary to OM/OI is generally considered good to excellent if rapid surgical decompression and/or debridement is performed.^1^, ^3^ It is important to obtain a rapid diagnosis to enable appropriate treatment as acute deterioration is possible, as described in one dog with rupture of an intracranial abscess and subsequent herniation.^3^ In two dogs, it was elected to continue medical management due to improving neurological status, financial constraints and/or owner consideration of co‐morbidities (including one dog having undergone cervical surgery 3 days previously). From follow‐up, persistent vestibular signs (mild head tilt and ataxia) were reported despite prolonged antibiotic therapy, with one dog reportedly neurologically normal following 8 weeks of treatment. In six dogs that underwent surgical management of intracranial empyema of varying aetiology in a previous study,^1^ all were deemed neurologically normal within 2 weeks. Hence, surgical treatment may lead to quicker resolution of clinical signs without evidence of recurrence. Based on our study and previous reports, surgery seems to be the treatment option of choice. However, given our small sample size, comparative statistical analysis regarding treatment was not performed. Although the surgery does not directly address the associated meningitis/meningoencephalitis, by debriding the infectious source, dogs may show a quicker resolution of clinical signs in a procedure with fairly minimal complications.

This case series describes 10 French bulldogs with a diagnosis consistent with bacterial meningitis or meningoencephalitis secondary to suspected intracranial extension of OM/OI. We suggest that French bulldogs could be at greater risk of developing this condition due to poor auditory tube drainage and underlying dermatological disease. Bacterial meningitis/meningoencephalitis may be more prevalent than previously thought within the breed and should be included as a differential diagnosis in dogs presenting with vestibular dysfunction and/or pain localised to the cervical or aural region. The outcome with prompt therapy can be positive, with full resolution of clinical signs possible.

## AUTHOR CONTRIBUTIONS

Sarah Butterfield was responsible for the case management, data collection and original draft of the manuscript. Danielle Whittaker, Joana Tabanez and Jordina Caldero Carrete also contributed to case management and data collection. Clare Pitchford and Charles R.J. Mattias examined the cytology samples and provided images included in the study. Danielle Whittaker, Abbe Crawford and Clare Rusbridge contributed to case management, supervised the project and reviewed the final manuscript.

## CONFLICT OF INTEREST STATEMENT

The authors declare they have no conflicts of interest.

## FUNDING INORMATION

The authors received no specific funding for this work.

## ETHICS STATEMENT

This was a case‐based retrospective study of clinical cases and so formal ethical approval was not needed.

## Data Availability

The authors provided all the data required for the study. Any additional information required may be provided upon authors request.

## References

[1] Forward AK , Plessas IN , Guilherme S , De Decker S . Retrospective evaluation of the clinical presentation, magnetic resonance imaging findings, and outcome of dogs diagnosed with intracranial empyema (2008–2015): 9 cases. J Vet Emerg Crit Care (San Antonio). 2019;29:431–8. 10.1111/vec.12859 31218823

[2] Spangler E , Dewey C . Meningoencephalitis secondary to bacterial otitis media/interna in a dog. J Am Anim Hosp Assoc. 2000;36:239–43. 10.5326/15473317-36-3-239 10825096

[3] Sturges BK , Dickinson PJ , Kortz GD , Berry WL , Vernau KM , Wisner ER et al. Clinical signs, magnetic resonance imaging features, and outcome after surgical and medical treatment of otogenic intracranial infection in 11 cats and 4 dogs. J Vet Intern Med. 2006;20:648–56. 10.1892/0891-6640(2006)20[648:csmrif]2.0.co;2 16734103

[4] Moore SA , Bentley RT , Carrera‐Justiz S , Foss KD , da Costa RC , Cook LB . Clinical features and short‐term outcome of presumptive intracranial complications associated with otitis media/interna: a multi‐center retrospective study of 19 cats (2009–2017). J Feline Med Surg. 2019;21:148–55. 10.1177/109861218764582 29667535 PMC10814610

[5] Kangsanarak J , Navacharoen N , Fooanant S , Ruckphaopunt K . Intracranial complications of suppurative otitis media: 13 years’ experience. Am J Otol. 1995;16:104–9. 8579165

[6] Neely JG . Intratemporal and intracranial complications of otitis media. In: Bailey BJ , editor. Head and neck surgery—otolaryngology. Philadelphia, PA, USA: JB Lippincott Co; 1993. p. 1607–22.

[7] Albers FW . Complications of otitis media. The importance of early recognition. Am J Otol. 1999;20:9–12. 9918164

[8] Schwaber MK , Pensak ML , Bartels LJ . The early signs and symptoms of neurotologic complications of chronic suppurative otitis media. Laryngoscope. 1989;99:373–5. 10.1288/00005537-198904000-00002 2927213

[9] De Lahunta A , Glass E , Kent M . Veterinary neuroanatomy and clinical neurology. 4th ed. Philadelphia, PA, USA: WB Saunders; 2014;343–67, 431 47.

[10] Lorek A , Dennis R , Van Dijk J , Bannoehr J . Occult otitis media in dogs with chronic otitis externa—magnetic resonance imaging and association with otoscopic and cytological findings. Vet Dermatol. 2020;31:146–53. 10.1111/vde.12817 31858646

[11] Gotthelf L . Diagnosis and treatment of otitis media in dogs and cats. Vet Clin North Am Small Anim Pract. 2004;34:469–87. 10.1016/j.cvsm.2003.10.007 15062620

[12] Foster A , Morandi F , May E . Prevalence of ear disease in dogs undergoing multidetector thin‐slice computed tomography of the head. Vet Radiol Ultrasound. 2015;56:137–43. 10.1111/vru.12180 25046431

[13] Wolf D , Lüpke M , Wefstaedt P , Klopmann T , Nolte I , Hermann S . Optimising magnetic resonance image quality of the ear in healthy dogs. Acta Vet Hung. 2011;59:552–8. 10.1556/AVet.59.2011.1.5 21354941

[14] Hygino da Cruz LCJ , Domingues RC . Intracranial infection. In: Atlas SW, editor. Magnetic resonance imaging of the brain and spine. Vol 1. 4th ed. Philadephila, PA, USA: Lippincott Williams and Willkins; 2009. p. 929–1025.

[15] Cole LK , Kwochka KW , Kowalski JJ , Hillier A . Microbial flora and antimicrobial susceptibility patterns of isolated pathogens from the horizontal ear canal and middle ear in dogs with otitis media. J Am Vet Med Assoc. 1998;212:469–87. 9491161

[16] Lopes MG , Soares FO , Alves EGL , Rosado IR . Otitis media and internal with brainstem extension in a French bulldog. Acta Sci Vet. 2021;49(Suppl 1):716. 10.22456/1679-9216.109615. Available from: https://seer.ufrgs.br/index.php/ActaScientiaeVeterinariae/article/view/109615/pdf. Accessed 5 Apr 2023.

[17] Salgüero R , Herrtage M , Holmes M , Mannion P , Ladlow J . Comparison between computed tomographic characteristics of the middle ear in nonbrachycephalic and brachycephalic dogs with obstructive airway syndrome. Vet Radiol Ultrasound. 2016;57:18–24. 10.1111/vru.12337 26765680

[18] Mielke B , Lam R , Ter Haar G . Computed tomographic morphometry of tympanic bulla shape and position in brachycephalic and mesaticephalic dog breeds. Vet Radiol Ultrasound. 2017;58:53–68. 10.1111/vru.12529 28726244

